# Synchronous multifocal necrotizing fasciitis prognostic factors: a retrospective case series study in a single center

**DOI:** 10.1007/s15010-016-0932-9

**Published:** 2016-10-24

**Authors:** Ching-Yu Lee, Yen-Yao Li, Tsan-Wen Huang, Tsung-Yu Huang, Wei-Hsiu Hsu, Yao-Hung Tsai, Jou-Chen Huang, Kuo-Chin Huang

**Affiliations:** 1 Department of Orthopedic Surgery, Chang Gung Memorial Hospital, No. 6, West Sec., Jiapu Road, Puzi City, Chiayi 613 Taiwan; 2 Division of Infectious Diseases, Department of Internal Medicine, Chang Gung Memorial Hospital, Chiayi, Taiwan; 3 Graduate Institute of Clinical Medical Sciences, College of Medicine, Chang Gung University, Taoyuan, Taiwan; 4 Department of Ophthalmology, Chang Gung Memorial Hospital, Chiayi, Taiwan; 5 College of Medicine, Chang Gung University, Taoyuan, Taiwan

**Keywords:** Synchronous, Multifocal, Necrotizing fasciitis, Prognostic factors

## Abstract

**Purpose:**

No reports have been published on synchronous multifocal necrotizing fasciitis (SMNF), a multifocal presence of necrotizing fasciitis in different extremities. We evaluated the clinical characteristics and outcomes of SMNF.

**Methods:**

Eighteen patients (14 men, 4 women; mean age: 59 years) diagnosed with SMNF of the extremities between January 2004 to December 2012 were enrolled and evaluated.

**Results:**

*Vibrio* species were the most commonly (78%; *n* = 14) isolated; others were two cases (11%) of *Aeromonas* spp., one case (6%) of group A β-hemolytic streptococcus, and one case of coagulase-negative *staphylococcus*. SMNF was in the bilateral lower limbs (72%; *n* = 13), bilateral upper limbs (17%; *n* = 3), and one patient with one upper and one lower limb (11%). Non-surviving patients had more bilateral lower limb involvement and thrombocytopenia.

**Conclusions:**

Most patients with SMNF were male and had bilateral lower limb and marine Gram-negative bacteria involvement. The mortality of SMNF remained extremely high in patients with involvement of bilateral lower limb and initial thrombocytopenia.

## Introduction

Necrotizing fasciitis (NF) is an uncommon deep soft tissue infection characterized by a rapidly spreading necrosis of superficial fascia and subcutaneous tissue [1]. Although typical NF involves primarily a single site, some recent studies report the concurrent involvement of multiple sites [2–4]. Some reports [2, 5, 6] incidentally indicate that multifocal necrotizing fasciitis leads to a higher mortality rate than does monofocal NF. We found no reports that focused primarily on synchronous multifocal necrotizing fasciitis (SMNF). Thus, we evaluated the clinical characteristics and outcomes of SMNF.

## Patients and methods

### Patients

We reviewed the records of patients diagnosed with and treated for SMNF at our hospital from January 2004 to December 2012. SMNF was defined as a multifocal presence of necrotizing fasciitis (NF) in different extremities at the initial visit. It was defined using surgical findings: the presence of grayish necrotic skin, subcutaneous fat and fascia, no resistance of normally adherent fascia to digital blunt dissection, and a purulent discharge resembling foul-smelling dishwater. Histopathological tissue specimens were obtained to confirm the diagnoses. Exclusion criteria were: (1) monofocal necrotizing fasciitis, (2) central necrotizing fasciitis (involvement of trunk or perineum), (3) a negative microbiological culture of the infected specimen, (4) concomitant chronic infections (e.g., osteomyelitis), and (5) a preexisting chronic wound (e.g., diabetic foot ulcer and decubitus ulcer).

### Treatment protocol

The treatment protocol included adequate antibiotic therapy and prompt radical debridement. All patients underwent emergency surgery within 1 h of admission when necrotizing fasciitis was suspected. Patients with multiple comorbidities, or an unstable hemodynamic status (systolic blood pressure <90 mmHg or mean arterial pressure <65 mmHg) were transferred to the intensive care unit for postoperative intensive care. Surgical debridement was done every other day if progressive necrotic changes combined with a deteriorating clinical presentation. Initial empiric broad-spectrum antibiotics were administered and adjusted based on blood and tissue culture results. Soft tissue reconstruction was done until the infected necrotic tissue was controlled and stabilized.

### Data analysis

Patient characteristics, presenting signs and symptoms, location of infection, underlying comorbidities, laboratory findings at the time of admission, bacteriologic results, and final outcomes in all patients were reviewed using the electronic database at our hospital. In regard to laboratory analysis, leukocytosis was defined as a white blood cell count greater than 12,000/μl, and thrombocytopenia was defined as a platelet count lower than 150,000/μl of blood. To assess clinical outcomes after antibiotic therapy and surgical debridement, mortality was defined as death because of progressive sepsis or medical complications within 12 months after surgery. To evaluate the predictive factors of mortality in SMNF, the variables were analyzed between survivors and non-survivors.

### Statistical methods

Univariate analysis was used to determine factors associated with the survivors and non-survivors. An independent Student’s *t* test was used for numerical data. A *χ* ^2^ analysis or a Fisher’s exact test was used for categorical data. Descriptive data are presented as the mean with standard deviation for quantitative variables and as frequency for categorical variables. Statistical significance was set at *p* < 0.05. SPSS 12.0 for Windows (SPSS Institute, Chicago, IL, USA) was used for all statistical analyses.

## Results

### Patient characteristics

We reviewed 401 patients diagnosed with NF at our hospital from January 2004 to December 2012. We excluded 383 patients: 150 with monofocal NF of the extremities, 88 with NF involving the trunk, 128 with concomitant chronic osteomyelitis, surgical wound infection, chronic diabetic or decubitus ulcer, and 17 with culture-negative NF. Eighteen (5%) patients (14 men, 4 women; mean age 59 years; age range 38–79 years) (Table 1) were diagnosed with SMNF of the extremities. The survivors were followed up for a minimum of 1 year (mean 16 months; range 12–24 months). *Vibrio* species [14 (78%) cases] were the most common. Others were *Aeromonas* spp. [2 (11%) cases], group A β-hemolytic *streptococcus* [1 (6%)], and coagulase-negative *staphylococcus* [1 (6%)]. Twelve (67%) of the 18 enrolled patients presented in shock on admission and required aggressive resuscitation. The extremity distribution of NF included the bilateral lower limbs (72%), bilateral upper limbs (17%), and one patient with one upper and one lower limb (11%) (Table 2). Fourteen patients immediately underwent a fasciotomy and debridement, and four patients (22%) had an initial amputation. Twelve patients with SMNF died: ten patients died of the uncontrolled original infection, one patient died of complicated pneumonia and a fungal urinary tract infection with fungemia, and one patient died of comorbidities [liver cirrhosis (Child-Pugh Class C) and upper gastrointestinal bleeding] within 4 months after hospital discharge.

**Table 1:** Patient characteristics

PT #	Age (years)	Sex	Occupation	Microbe	Comorbidity	S/S at first visit	Exposure history
1	38	Male	Fisherman	*V. vulnificus*	LC, AA	S, H, P	Seawater
2	38	Male	Farmer	β-hem. strep.	LC, HBV, HCV, AA	S, F, P	NA
3	58	Male	Fisherman	*V. vulnificus*	NA	F, H, P	Seawater
4	70	Male	Fisherman	*V. vulnificus*	DM	S, H, P	Seawater
5	76	Male	Fisherman	*CoNS*	AA, COPD	P	NA
6	59	Male	Plasterer	*V. vulnificus*	AA, AR, IE	P	Fish
7	60	Male	Fisherman	*V. vulnificus*	LC, AA, ESRD	S, H, P	Seawater
8	79	Male	Farmer	*V. vulnificus*	ESRD, gout	S, H, P	Farmland
9	52	Female	NA	*V. vulnificus*	LC, HBV, HCV, HCC	S, F, P	NA
10	57	Male	Plasterer	*V. vulnificus*	HCV, DM	S, F, P	Oyster
11	63	Female	Barber	*V. vulnificus*	LC, HCV	S, F, H, P	NA
13	51	Male	NA	*V. cholerae*	LC, HBV, ESRD, AA	S, F, H, P	Seafood
14	59	Male	Farmer	*A. sobria* *Klep. pneumoniae*	LC, HCC, AA	S, P	NA
15	53	Male	Plasterer	*V. vulnificus*	LC, AA	H, P	NA
16	79	Male	Farmer	*V. vulnificus*	DM, AA, AR	S, F, H, P	NA
17	68	Female	Farmer	*A. hydrophila*	Steroid abuse	S, H, P	Bamboo stabbing
18	64	Female	Barber	*V. vulnificus*	HCV, DM, AR	S, F, H, P	NA

*PT* patient, *S/S* signs and symptoms, *V. Vibrio*, *CoNS* coagulase-negative staphylococci, *A.* *Aeromonas*, *β-hem. Strep.* group A β-hemolytic streptococcus, *Klep. Klebsiella, AA* alcohol abuse, *LC* liver cirrhosis, *HBV* hepatitis B virus, *HCV* hepatitis C virus, *HCC* hepatic cell carcinoma, *DM* diabetes mellitus, *COPD* chronic obstructive pulmonary disease, *ESRD* end-stage renal disease, *AR* aortic regurgitation, *S* shock, *F* fever, *H* hemorrhagic bullae, *P* limb pain, *NA* not available

**Table 2:** Additional patient characteristics

PT #	Affected limbs	Onset day	Antibiotics	Initial surgery	Outcome
1	RF, LF	3	Ceftriaxone	FD	Survival
2	RA, LT	2	Ceftriaxone	FD	Survival
3	RH, LH	1	Imipenem & cilastatin sodium, MSD	FD	Survival
4	RH, RH	2	Ceftriaxone	FD	Survival
5	RA, LA	5	Ceftriaxone	FD	Survival
6	RH, LL	1	Ceftriaxone	AMP, FD	Survival
7	RL, LL	1.5	Ceftriaxone	FD	Death
8	RL, LL	1	Imipenem & cilastatin sodium, MSD	FD	Death
9	RL, LL, LH	1	Ceftriaxone	FD	Death
10	RL, LL	1	Ceftriaxone	Bil. AMP	Death
11	RL, LL	1	Ceftriaxone	FD	Death
12	RL, LL	1	Ceftazidime	Bil. AMP	Death
13	RL, LL	1	Ceftriaxone	FD	Death
14	RL, LL	1	Ceftriaxone	FD	Death
15	RL, LF	1	Ceftriaxone	FD	Death
16	RL, LL	1	Ceftazidime	FD	Death
17	RL, LL	1	Ceftriaxone	FD	Death
18	RH, LH	1	Ceftriaxone	Bil. AMP	Death

*PT* patient, *RA* right arm, *LA* left arm, *RH* right hand, *LH* left hand, *RL* right leg, *LL* left leg, *RF* right foot, *LF* left foot, *FD* fasciotomy and debridement, *AMP* amputation, *Bil. AMP* bilateral amputation

### Patient characteristics compared

Almost all of the non-survivors developed NF of the bilateral lower limbs, except for one non-survivor with affected bilateral upper limbs; all of the survivors had NF of the bilateral upper limbs (*p* = 0.005) (Table 3). Differences in age, gender, comorbidities, and duration of symptoms were not significant.

**Table 3:** Comparison of patient characteristics

Characteristic	Survivors (*n* = 6)	Non-survivors (*n* = 12)	*p*
Age (years)	57 ± 16	61 ± 10	0.486
Males	6 (100)	8 (67)	0.245
Duration of symptoms (days)	2.3 ± 1.5	1 ± 0.1	0.09
Extremities affected			0.005*
Bilateral upper	3 (50)	1 (8)	
Bilateral lower	1 (17)	11 (92)	
Both upper and lower	2 (33)	0	
Comorbidity			
Diabetes mellitus	1 (17)	2 (17)	1
ESRD	0	3 (25)	0.515
CLD	2 (33)	9 (75)	0.141
Liver cirrhosis	2 (33)	7 (58)	0.31
Hepatitis B virus or hepatitis C virus	1 (17)	6 (50)	0.152

Data are mean ± standard deviation or number (%)

*CLD* chronic liver diseases: liver cirrhosis, hepatitis B virus, or hepatitis C virus

* *p* < 0.05

### Laboratory data compared

Survivors had a higher rate of leukocytosis than did non-survivors (83 vs. 17%, *p* = 0.013) (Table 4). The prevalence of thrombocytopenia was higher in non-survivors than in survivors (92 vs. 17%, *p* = 0.004). Bacteremia occurred in 50% of survivors and in 83% of non-survivors (*p* = 0.268). The levels of serum hemoglobin, C-reactive protein, and albumin were not significantly different between the two groups.

**Table 4:** Comparison of laboratory data

Laboratory data	Survivors (*n* = 6)	Non-survivors (*n* = 12)	*p*
Leukocytosis (≥12,000/μL)	5 (83)	2 (17)	0.013^*^
Thrombocytopenia	1 (17)	11 (92)	0.004^*^
Hemoglobin (g/dL)	14.0 ± 1.8	13 ± 1.6	0.229
C-reactive protein (mg/dL)	198.0 ± 119	61 ± 40	0.061
Albumin (g/dL)	2.4 ± 0.5	2 ± 0.6	0.235
Bacteremia	3 (50)	10 (83)	0.268

*p < 0.05

### Bacterial cultures compared

Gram-negative bacteria were predominantly isolated in both groups, especially *Vibrio vulnificus*. There was no significant difference in bacterial species between the groups (Table 5).

**Table 5:** Comparison of bacterial cultures from tissue specimens

Bacterial culture	Survivors (*n* = 6)	Non-survivors (*n* = 12)	*p*
Gram-positive bacteria	2 (33)	0	0.098
Gram-negative bacteria	4 (67)	12 (100)	
Group A β-hemolytic strep.	1 (17)	0	
Coagulase-negative staph.	1 (17)	0	
*Vibrio vulnificus*	4 (67)	9 (75)	
*Vibrio cholerae*	0	1 (8)	
*Aeromonas sobria*	0	1 (8)	
*Aeromonas hydrophila*	0	1 (8)	
*Klep. pneumoniae*	0	1 (8)	

*strep.* streptococcus, *staph.* staphylococcus, *Klep. Klebsiella*

## Discussion

This is the first report of a case series of SMNF and analysis of its predictive factors for mortality. In the current study, SMNF accounted for 5% of NF. The main characteristics of our patients with SMNF were male sex, lower limb involvement, and marine Gram-negative bacterial infection. The incidence of SMNF in noncontiguous sites is not clear in the literature. Park et al. [5], in a retrospective case review of 217 patients with NF in the coastal area of South Korea, reported that at least 73 (34%) patients had noncontiguous multifocal involvement with highly frequent isolation of Gram-negative marine bacteria. El-Khani et al. [2], in a case report and systematic review of 33 cases of multifocal NF, reported that most patients were male, that their lower limbs were involved, and that there were 18 Gram-positive bacteria, 6 Gram-negative bacteria, 5 anaerobic bacteria, 1 yeast, and 4 polymicrobial isolates in the microbiologic cultures. Similar to the findings of Park et al. [5], most of our cases were classified as type III NF (infection by monomicrobial pathogen, usually Gram-negative bacteria). Moreover, in 89% of our patients with SMNF, marine bacteria—*Vibrio* spp. in 78% and *Aeromonas* spp. in 11%—were most commonly isolated. In contrast, there were 12% type I NF (infection by polymicrobial bacteria) and 52% type II (infection by a monomicrobial pathogen, usually Gram-positive bacteria) in El-Khani et al. [2]. The different bacteria isolated might be attributable to the different geographical regions and epidemiology of the cases reported [7–9]. Marine bacteria (e.g., *Vibrio* spp. and *Aeromonas* spp.) are often found in the warm coastal areas of Asia (e.g., Taiwan, Hong Kong, South Korea, Singapore, and Thailand), the Gulf of Mexico, South America, and Australia [10–12]. Also, NF in patients with chronic liver diseases is usually a monomicrobial infection, caused primarily by Gram-negative bacteria [13, 14]. Our hospital is in the Tropic of Cancer in southwestern Taiwan, where many local inhabitants work in fisheries or consume seafood, and where alcoholism, chronic viral hepatitis, and liver cirrhosis are highly prevalent. Therefore, a specific epidemiology, i.e., a high prevalence of chronic liver diseases in a coastal area, would make marine Gram-negative bacteria the main causative pathogens of SMNF.

The overall mortality rate of our patients with SMNF was 67%. Most of the literature focuses only on monofocal NF and reports a cumulative average mortality rate of 34%. The survival rate for NF patients has risen to as high as 85% as the awareness of monofocal NF has increased and as more-frequent aggressive treatment and adjuvant therapy for the disease have increased. In contrast, multifocal NF remains highly fatal. Park et al. [5] reported that patients with infections in multiple sites had a mortality rate of 62%, significantly higher than the 30% in patients with a single-site infection. Similarly, Hua et al. [6], in a retrospective single-center cohort study, said that multifocal necrotizing soft tissue infection was an independent risk factor for mortality. SMNF might be caused by rapid metastatic septic embolization or simultaneous multifocal inoculation and the subsequent development of a necrotizing infection. The former implies fulminant septicemia with the hematogenous spread of bacteria, and the latter implies that more than one site has been concomitantly exposed to pathogenic bacteria. Both conditions can lead to overwhelming sepsis. Also, a large area of skin, subcutaneous tissue, fascia, and underlying necrotic destruction of muscle in multiple-site necrotic deep soft tissue will cause extensive complications and a high mortality rate. Like extensive NF that involves a large surface area of the body [15], an immediate fasciotomy and radical removal of necrotic tissue without a misdiagnosis is important when treating SMNF. The treatment protocol should consist of prompt antibiotic therapy, prolonged intensive care, nutritional support, serial surgical debridement, and reconstruction of soft tissue to reduce mortality and morbidity rates.

**Fig. 1:**
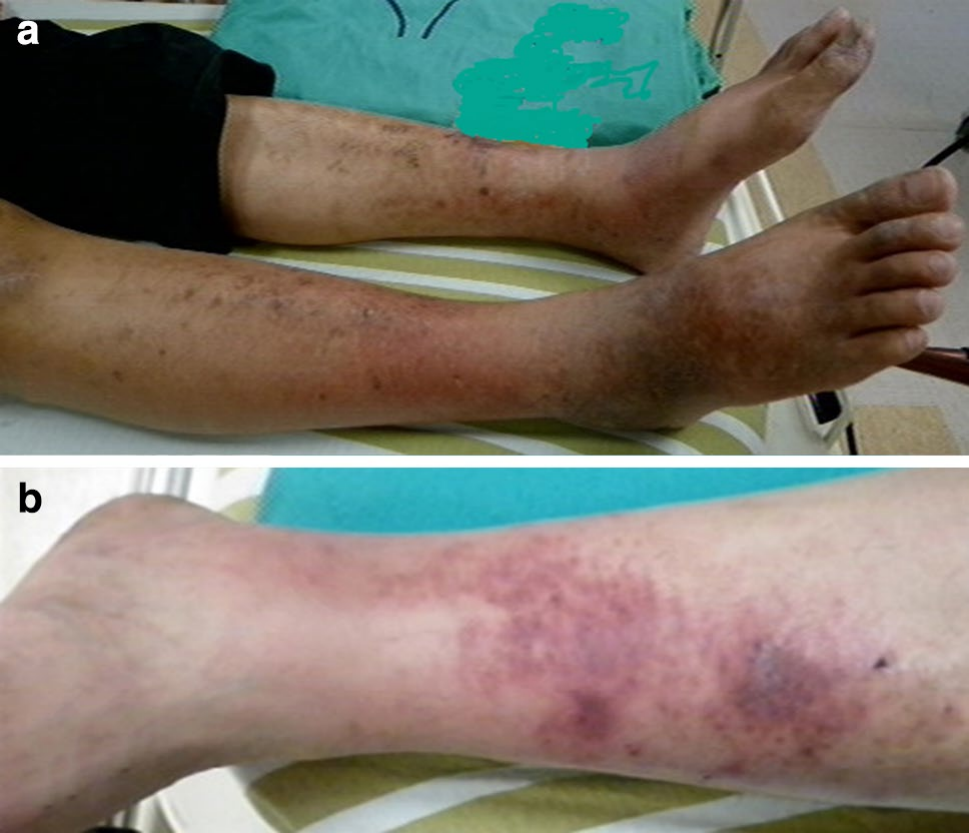
Extrahepatic cutaneous dermatitis and vasculitis in patient number 13. **a** Dermatitis with hyperpigmentation-affected bilateral lower limbs; **b** non-blanching purpura were the main characteristics of vasculitis

**Fig. 2:**
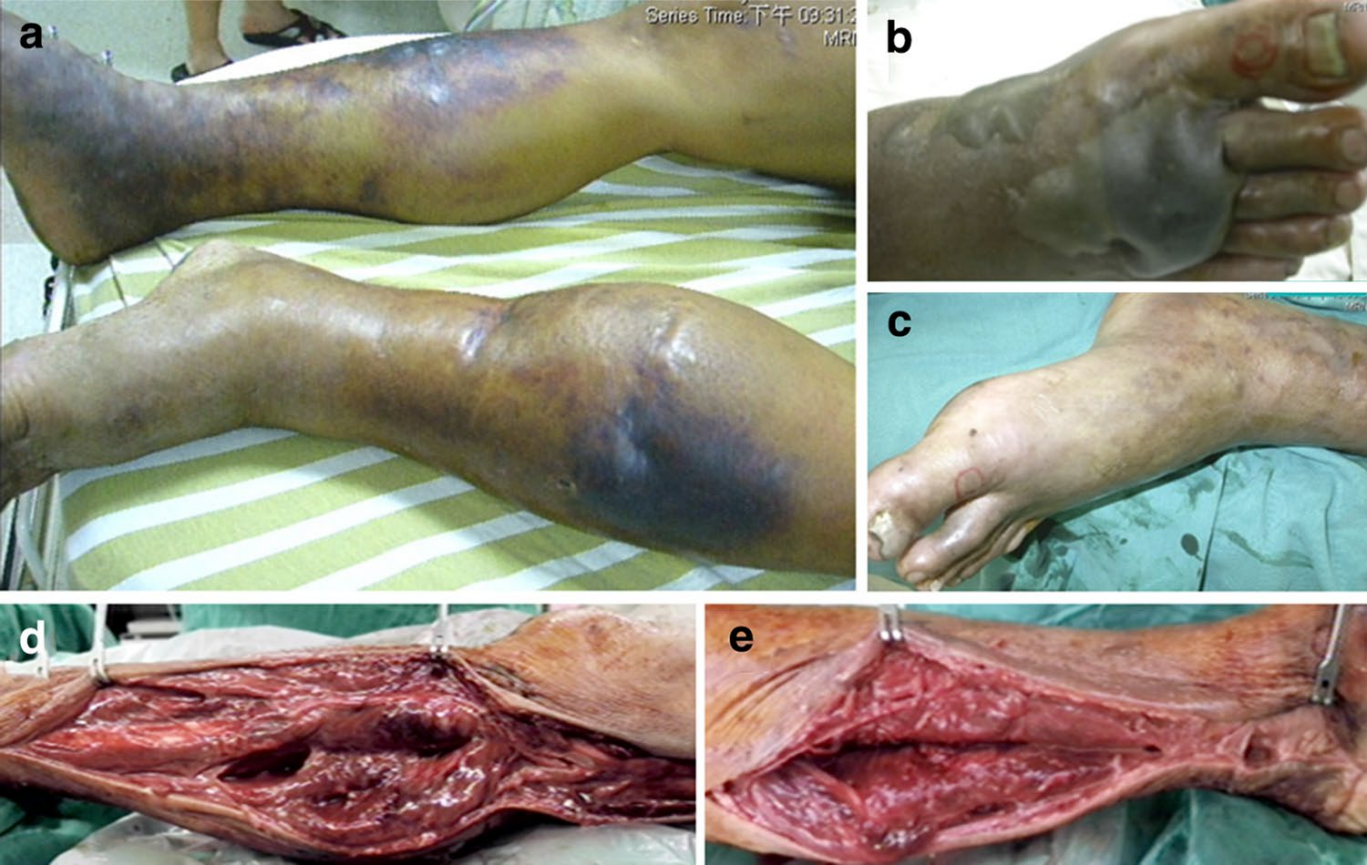
Multifocal necrotizing fasciitis 5 years after the onset of extrahepatic cutaneous lesions in patient number 13. **a** A multifocal presence of necrotizing fasciitis occurred in the bilateral lower limbs; **b** hemorrhagic bullae developed on the dorsum of the right foot; **c** Violet, swollen skin with out-of-proportion pain was rapidly progressive in the left leg; **d** and **e** there was no resistance of normally adherent fascia to digital blunt dissection, and there was a purulent discharge resembling foul-smelling dishwater in immediate fasciotomies on the bilateral lower legs

In the present study, non-survivors had a higher prevalence of involved bilateral lower limbs and thrombocytopenia than did survivors. There is a paucity of reports on the risk factors for SMNF mortality. However, the mortality rate in cases of monofocal NF with upper extremity involvement is not significantly different from that in cases with lower extremity involvement [16, 17]. El-Khani et al. [2] reported a significantly higher mortality rate in bilaterally affected lower limbs than in bilaterally affected upper limbs (60 vs. 0%), but the clinical mechanism of mortality related to lower extremity involvement is unclear. In the present study, chronic liver diseases were the most common comorbidities (61%) in SMNF, especially in non-survivors (75%). Some evidence [18–20] indicates that atherosclerosis and cutaneous vasculitis in the lower limbs were highly associated with chronic liver diseases (e.g., viral hepatitis and alcoholic hepatitis). Also, extrahepatic cutaneous vasculitis [21], primarily of the lower limbs, leads to cutaneous lesions and to an insufficient barrier against bacterial invasions (Fig. 1). Atherosclerosis, vasculitis, and devitalized soft tissue are the results of a poor blood supply, which causes the clinical deterioration of necrotizing deep soft tissue infection [22]. These comorbidities might result in a higher prevalence of involved bilateral lower limbs in patients who die from NF (Fig. 2).

Consistent with our findings for multifocal NF, thrombocytopenia has also been a predictive factor for mortality in monofocal necrotizing deep soft tissue [23, 24]. Physicians should be aware of the grave prognosis for patients with SMNF of the bilateral lower limbs and who present with thrombocytopenia. Compared with survivors, non-survivors tended to have lower, but not significantly different, C-reactive protein (CRP) levels. In contrast, Park et al. [5] reported that the CRP level was one predictor of mortality in NF. Using the CRP level to predict mortality is controversial. Many studies [25, 26] have reported that CRP levels are not significantly different between survivors and non-survivors. Moreover, CRP levels reach peak values between 48 and 72 h after the onset of bacterial infection [27], and CRP does not precisely indicate the severity of bacterial infections at a single time point. In the present study, non-survivors had a relatively shorter onset of symptoms, which might indicate a lower CRP level on admission.

This study has some limitations. This is a case series study with a small number of patients, because NF is a rare disease and our inclusion criteria were restricted to peripheral NF (involvement of extremities). Central NF (involving head, neck, abdomen, and perineum) was excluded, because its etiology and surgical procedures are not similar to those of peripheral NF. NF of the head and neck often arises from an odontogenic infection with mixed aerobic and anaerobic abscesses [28, 29]. NF of the abdomen or perineum might be the result of urogenital or colorectal infections by enteric microorganisms [30]. A colostomy is usually required when treating abdominal or perineal NF [31–33]. Moreover, we did not include preexisting chronic wounds, of which the etiology might be pyomyositis or osteomyelitis. Univariate analysis rather than multivariate analysis was used to determine predictive factors for mortality, because there were only a few patients in each group. Although this study indicated that the involvement of the bilateral lower limbs and thrombocytopenia were highly associated with mortality in SMNF, additional studies are needed to confirm this.

In conclusion, the incidence of SMNF of the extremities was 8% for all subtypes of NF. Male sex, bilateral lower limb involvement, and marine Gram-negative bacteria were the most frequently found factors in SMNF. The mortality of SMNF was as high as 67%, which was more frequently associated with initial thrombocytopenia and bilateral lower limb involvement. It is essential to detect a multifocal presence of NF in the extremities to justify an immediate fasciotomy and the radical removal of necrotizing tissue after prompt antibiotic therapy and sufficient resuscitation.

## Abbreviations

SMNF: Synchronous multifocal necrotizing fasciitis
NF: Necrotizing fasciitis
